# Isolation and evaluation of endophytic *Bacillus tequilensis* GYLH001 with potential application for biological control of *Magnaporthe oryzae*

**DOI:** 10.1371/journal.pone.0203505

**Published:** 2018-10-31

**Authors:** Hui Li, Ying Guan, Yilun Dong, Lu Zhao, Songhao Rong, Wenqian Chen, Miaomiao Lv, Hong Xu, Xiaoling Gao, Rongjun Chen, Lihua Li, Zhengjun Xu

**Affiliations:** Rice Research Institute of Sichuan Agricultural University, Chengdu, China; Fujian Agriculture and Forestry University, CHINA

## Abstract

Biological control is a promising measure in the control of plant disease. In the present study, we isolated 13 endophytic strains from *Angelica dahurica*. Among them, an endophytic strain which was named GYLH001 exhibited remarkable activity against *Magnaporthe oryzae*. 16S rRNA sequence analysis, biochemical and physiological proved that it is *Bacillus tequilensis*. The sterilized culture filtrate of GYLH001 can inhibit the growth of *M*.*oryzae*, which suggests the presence of secondary metabolites. Proved by experiment, GYLH001 can produce cellulase, protease, gelatinase, indole-3-acetic acid and 1-amino-cyclopropane-1-carboxylate deaminase. In addition, the temperature experiment showed that secondary metabolites produced by GYLH001 had good thermal stability. They can remain activity even heated at 100°C for 30 min. They also had good acid-resistance in heavily acidic condition. But under alkaline condition, the antifungal effect decreased significantly. By simulative field tests, the spraying of GYLH001 spore solution could prevent and treat rice blast. Through continuous separation and purification of sterilized culture filtrate and identification by mass spectrometry, the molecular weight of an active substance is 364.26. In the control of rice blast, *B*. *tequilensis* GYLH001 has potential as a biological control agent in agriculture.

## Introduction

Rice is the main staple food for a half of the world population [1]. With improvement of living standards, people have attached more importance to rice quality and yield [2]. However, losses caused by phytopathogenic fungi have caused great reduction of rice yield resulting in food shortage. Rice blast, caused by the ascomycete *Magnaporthe oryzae*, is a severe and highly popular disease that results in 10%-30% rice yield losses annually [3–5]. So, an increasing number of people have been planting resistant rice varieties and using chemical fungicide to prevent and treat rice blast effectively. Although chemical fungicide has many advantages like low cost and high effectiveness, overuse of chemical fungicide not only pollutes the environment but also makes rice blast become more drug resistance [1]. In addition, it is also hard to against rice blast by planting resistant rice varieties because of complexity of rice blast genetic background. Therefore, it is urgent to find more effective and safer methods to suppress rice blast. In recent years, there is an increasing interest of exploitation of rice blast for biological control by using plant beneficial microorganisms owing to their low toxicity and a lack of pathogen resistance [6]. *Bacillus* spp., actinomycetes, *Pseudomonas* spp. and other antifungal strains produce a lot of antibiotics and enzymes. Antibiotics are secondary metabolites which are produced by antagonistic microbes with diverse biological activities [5, 7–9]. And their use as biological control agents poses minimal detrimental effects on the environment [6].

Endophytic strains are living inside plant tissues without any obvious symptoms of infection or diseases for most of their life cycles and mutualistic to their hosts [10]. Endophytic strains are deemed to interact closely with their host plants and keep them from attacking by fungi, insects and mammals by secreting bioactive secondary metabolites to inhibit the growth of pathogens directly or improve resistance of host plants [11, 12]. As a matter of fact, endophytic strains have already reported that entophytic *B*. *subtilis* var. *amyloliquefaciens* EPB9, EPB10, EPCO29 and EPCO78 indicated significantly high inhibition of bacterial leaf blight [13]. Entophytic *B*. *subtilis* E1R-j exhibited high antifungal activity to *Gaeumannomyces graminis* var. *tritici* [14]. Besides, entophytic *B*. *subtilis* Lu144 effectively reduced disease of bacterial wilt of mulberry incidence [15]. However, there are few reports concerning *B*. *tequilensis* which indicated that it was closely related to *B*. *subtilis* isolated from *Angelica dahurica* as a biological control agent against pathogenic fungus [16].

*Angelica dahurica* is a traditional Chinese Medicine which has been widely used in China [17]. As an important herbal medicine, it shows strong antioxidant activity to treat an antipyretic and analgesic for cold, headaches, coryza, hypertension, and toothaches [18]. A lot of previous studies have been researched about isolation and identification of different constituents, for instance phthalides, organic acids, polysaccharides, polyacetylenes, amino acids, and trace elements [19]. Few studies have been indicated endophytic strains with their antifungal activity in *Angelica dahurica*.

In our laboratory, a lot of endophytic strains from *Angelica dahurica* had been isolated and screened *in vitro* on their antagonistic activities against *M*.*oryzae*. In this study, an endophytic bacteria displaying a strong inhibition against *M*.*oryzae* has been selected and named GYLH001, which was identified by morphology, biochemical and physiological properties and 16S rRNA sequence analysis. Furthermore, we designed a series of preliminary test to validate its potential value as a biological control agent.

## Materials and methods

### Isolation of endophytic strains

Endophytic strains had been separated from *Angelica dahurica*. All were collected without obvious symptoms of disease and the procedures of surface sterilization were described as Xiong et al [20]. All were cut into 10 mm in length and then were transferred into sterile mortar to be ground with sterile water. The paste of seeding was coated onto potato dextrose agar medium. After incubated for 2 to 3 days at 37°C in the incubator, strains appeared on the petri dish and were respectively isolated as a single colony on potato dextrose agar medium.

### Screening of the strong antagonistic strains against *M*.*oryzae*

Rice blast pathogenic fungus *M*.*oryzae* Guy11 [21] was provided by Plant Pathology Laboratory of Sichuan Agricultural University. To select the strong antagonist against *M*.*oryzae*, every strain was inoculated to homologous potato dextrose liquid medium to be incubated at 28°C, 180 rpm for 48 h in the rotary shaker. Every strain solution was taken out and stored in a small EP tube. 10 μL of sterilized culture filtrate of strains were taken out from every small EP tube and injected the edge of *M*.*oryzae* which had been deposited in the center of potato dextrose agar medium. And these medium were incubated at 28°C in the incubator. After incubated for 3 to 4 days, the inhibition of growth of *M*.*oryzae* was compared by observed the degree of dent. The strong antagonistic bacteria was named GYLH001 and taken for further study.

### Evaluation of antifungal activity

The inhibition of *M*.*oryzae* was tested: the strain GYLH001 was inoculated to potato dextrose liquid medium to be incubated at 28°C, 180 rpm in the rotary shaker. The fermentation broth was taken out at 48 h after inoculation and centrifuged at 10000 rpm at indoor temperature for 2 min to remove bacterial cells. The supernatant was filtered through 0.22 μm membrane filter and then the culture filtrate was mixed up with PDA at different concentrations (1:10, 1:20, 1:40, 1:80, 1:160, v/v). Last, the mixture was poured into Petri plates. The fungus cake of Guy11 were transferred to center of medium and incubated at 28°C in the incubator [22]. The *M*.*oryzae* colony diameter was measured after 7 days. The formula of relative inhibition rate was described as Sawai Boukaew [23].

Relativeinhibition(%)=[(R1−R2)/R1]×100

Where, R_1_ = *M*.*oryzae* colony diameter in the control and R_2_ = *M*.*oryzae* colony diameter in the dual culture plates with sterilized culture filtrate of GYLH001 mixed up with PDA.

### Identification of GYLH001 by 16S rRNA analysis and Biochemical and physiological test

The procedure of extracting of total genomic DNA were described as manufacturer’s instructions of a commercial DNA extraction kit. The 16S rRNA of the strain was amplified using forward primer (8-27F) 5´- AGAGTTTGATCCTGGCTCAG-3´ and reverse primer (1492R) 5´- GGTTACCTTGTTACGACTT -3´. The 50 μL mixture of PCR were placed in a small EP tube containing 2 μL of forward primer, 2 μL of reverse primer, 10 μL of 5×TransStart Fastplu fly Buffer, 1 μL of TransStart Fastplu Fly DNA Polymerase, 2 μL of the extracted DNA, 5 μL of 2.5 mM High pure dNTPs and 28 μL of sterile water. The polymerase chain reaction program was an initial denaturation for 5 min at 95 °C, an 36-cycle of denaturation (0.5 min at 95°C), annealing (0.5 min at 55°C) and extension (1.5 min at 72°C), and a final extension for 5 min at 72°C [24–26]. Purification of PCR products were used by a PCR Purification Kit and identification of PCR products were used by horizontal electrophoresis on a 1% agarosegel. The fragments of amplified 16S rRNA were transformed into *Escherichia coli*. Sequencing was sequenced by TSINGKE (Chengdu, China). Similarity of 16S rRNA sequences was compared by using the BLAST search program in the Ezbiocloud. (http://www.ezbiocloud.net)

The colony characteristics of strain GYLH001 were observed on PDA medium. According to Bergey’s Manual of Systematic Bacteriology, biochemical and physiological were test [27].

### The stability of pH and temperature of filtrate on the inhibition of growth of *M*. *oryzae*

The sterilized culture filtrate of GYLH001 was prepared and taken out of 70 mL to be respectively placed in the seven vitro and adjusted to various pH values to 1, 3, 5, 7, 11, 13 and stored in the refrigerator at 4°C. After 24 h, the samples were readjusted to pH 7 and taken out 1.5 mL from each of vitro to mix up with seven potato dextrose agar medium, which makes filtrate of concentration to 6.25 μL /mL (1:160, v/v). The fungus cake of Guy11 was transferred to petri dish. After incubation at 28°C for 7 days in the incubator, the growth of Guy11 was observed and the colony diameter of Guy11 was measured to assess growth inhibition rate compared to control petri dish without sterilized culture filtrate. This test set three repeats.

In the temperature test, the sterilized culture filtrate was exposed at 4°C, 28°C, 37°C, 60°C, 80°C, 100°C, 121°C for 30 min. when the sample had been cooled down to room temperature, the test could be done. The specific procedures were in accordance with pH tests.

The formula of relative inhibition rate was described above.

### Correlation between cell growth and antifungal activity

*B*.*tequilensis* GYLH001 was inoculated into 500 mL of potato dextrose liquid mediumin in a 1 L-flask and was incubated at 28°C, 180 rpm for 108 h. During incubation, some samples were taken out at intervals of 12 h for measuring OD_600_ and antifungal activity against *M*. *oryzae* Guy11 were test by measured the colony diameter of Guy11. The specific procedure was described above.

The formula of relative inhibition rate was described above.

### Determination of enzymes and metabolites produced by *B*. *tequilensis* GYLH001

The experiment of chitinase production was conducted as previously described [28]. The tests of cellulose, protease, Hydrocyanic acid and indole-3-acetic acid (IAA) production were conducted in accordance with Gopalakrishnan et al [29]. The method of test of gelatinase production was conducted as described by Bose et al [30]. Detection method of 1-amino-cyclopropane-1-carboxylate (ACC) deaminase was in accordance with Donna M. Penrose [31].

### Experiment on isolated leaves

The experiment was divided into prevention group, treatment group and control group. First, the 6-Benzylaminopurine solution 1 mg/mL was prepared and leaves of rice without obvious symptoms of disease were placed into petri dishes with 6-Benzylaminopurine solution. After that, every leaf was poked slightly by knife which was in favor of infection of *M*. *oryzae*.

Prevention group: a 10 μL volume of droplets of the sterilized culture filtrate of GYLH001 and bacterial suspension of GYLH001 were applied to slightly punctured sites of leaves. Then the leaves were incubated at 25°C in the dark. After 24 h, all punctured sites of leaves were inoculated with 10 μL droplets of spore suspension of *M*. *oryzae* (1 × 10^5^ spores/mL). The leaves was incubated at 25°C in the dark for 24 h. Subsequently, all were incubated in the light at room temperature [32]. In the control group, sterile water was used instead of the sterilized culture filtrate and other steps were unchanged. After 7 days, lesion diameter was compared.

Treatment group: the method was the same as prevention group, but leaves were inoculated with spore suspension of *M*. *oryzae* firstly.

### Experiment on living leaves

The experiment was finished under growth chamber conditions. The bioassay was conducted in accordance with previous methods [33]. Firstly, 30-day-old rice seedlings were transferred to the inoculation chamber. In the preventive experiment, the rice seedlings were sprayed to runoff with the sterilized culture filtrate of GYLH001 containing Tween 20. After 24 h, by spraying with a spore suspension (1 × 10^5^ spores/mL), the rice seedlings were inoculated with *M*. *oryzae*. Control group had been done by the following method (i, spraying spore suspension of *M*. *oryzae* after spraying the sterilized culture filtrate of GYLH001; k, spraying spore suspension of *M*. *oryzae* after spraying 0.75g / L tricyclazole; l, without any treatment).

### Isolation of the antifungal substances

After centrifugation at 8000 rpm at room temperature for 15 min, the supernatant (1L) was decompressed and steamed by a rotary vacuum evaporator (40°C, 30 mbar) and redissolved with methanol. After that, the concentrated solution was filtered by passing it at a constant flow rate through a column filled with SciBioChem Middle Chromatogram Isolated Gel (22mm^2^×459mm). The substances was eluted with gradient mixture of methanol and H_2_O and 4-fold to the column volume at a constant flow rate on the column. The activity fraction was concentrated by a rotary vacuum evaporator and redissolved with methanol. The crude extract was purified by HPLC (Inersil ODS-3, 0.8 mL/min, MeOH-H_2_O, 67:33).

The collected different fractions were steamed by a rotary vacuum evaporator and tested for antifungal activity. Then, the high activity fraction was chosen for further purification by HPLC (Waters C18, 0.8 mL/min, H_2_O-Acetonitrile, 57:43). The collected substances was detected by mass spectrometry.

### Statistical analysis

The experiments were repeated at least in three times. Statistical analysis of the data was evaluated with SPSS 20.0 software. The value of *p* < 0.05 was deemed to indicate statistical significance. All data was expressed as mean standard deviation.

## Result

### Isolation and screening of endophytic strains antagonistic to *M*. *oryzae*

**Fig 1:**
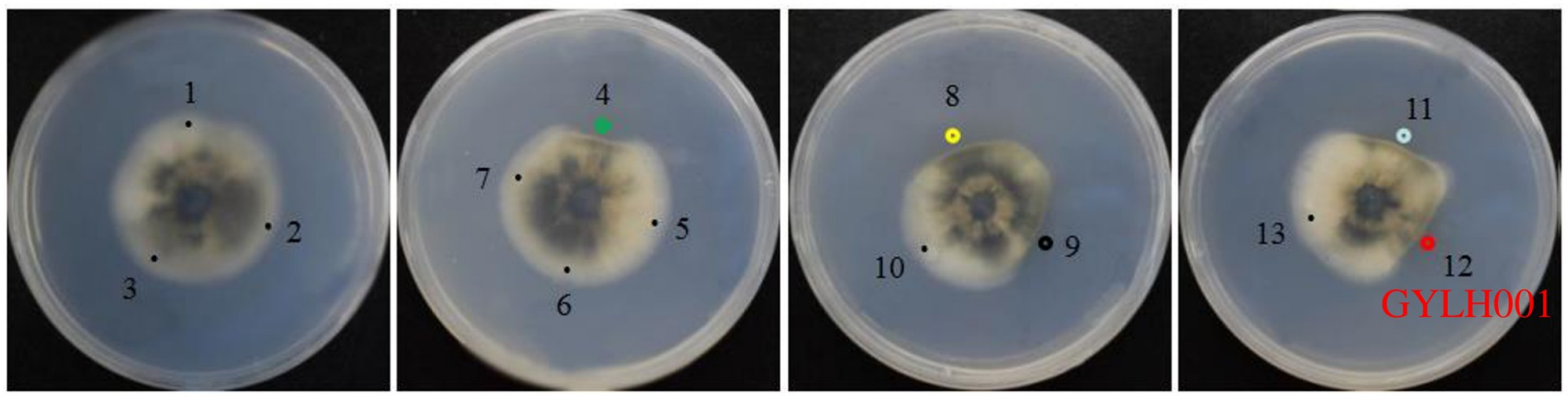
Screening of the strong antagonistic strains against *M*.*oryza*. Serial numbers 1 to 13 respectively represent 13 kinds of strains. The highlighted shows that these strains have activity against *M*. *oryzae*.

In order to seek out strains antagonistic to *M*. *oryzae* which resulted in severe reduction of rice yield, a total of 13 different colonies were isolated from *Angelica dahurica*. Five sterilized culture filtrate of different colonies displayed obvious antifungal activity against *M*. *oryzae* Guy11 with mycelia growth inhibition by test. GYLH001 exhibited particularly strong activity (Fig 1).

### Evaluation of antifungal activity

**Fig 2:**
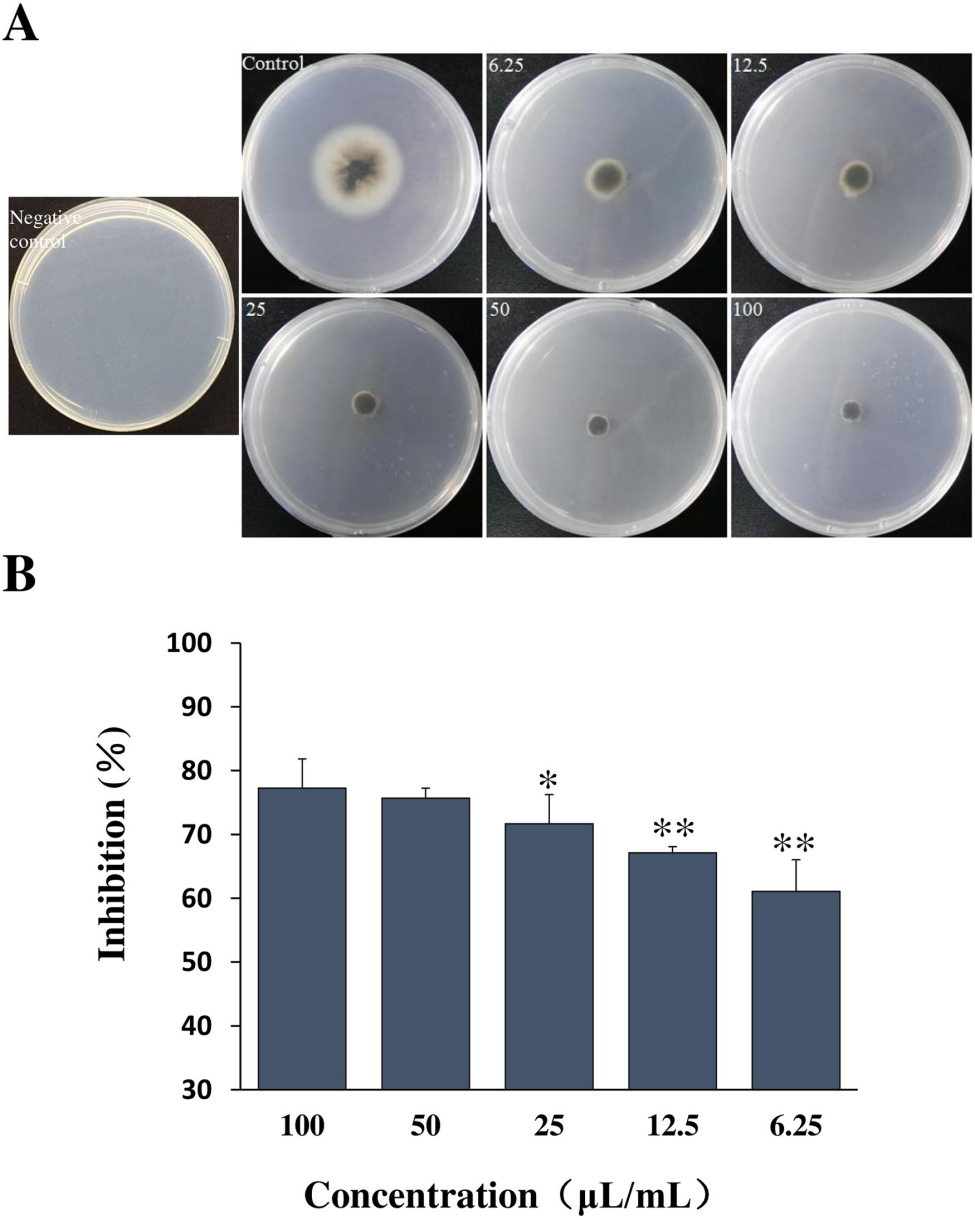
Effect of different concentrations on the inhibition of *M*. *oryzae*.

As shown in Fig 2, the sterilized culture filtrate of GYLH001 had a significant inhibition of growth of *M*. *oryzae*. With the concentration of the sterilized culture filtrate increased, the inhibitory activity was increased obviously. When the minimum concentration was 6.25μL/mL, the relative inhibitory rate was 61.07%. It could be seen that strain GYLH001 had a strong antagonistic on the *M*. *oryzae* and great research value.

### Identification of the strain GYLH001

**Fig 3:**
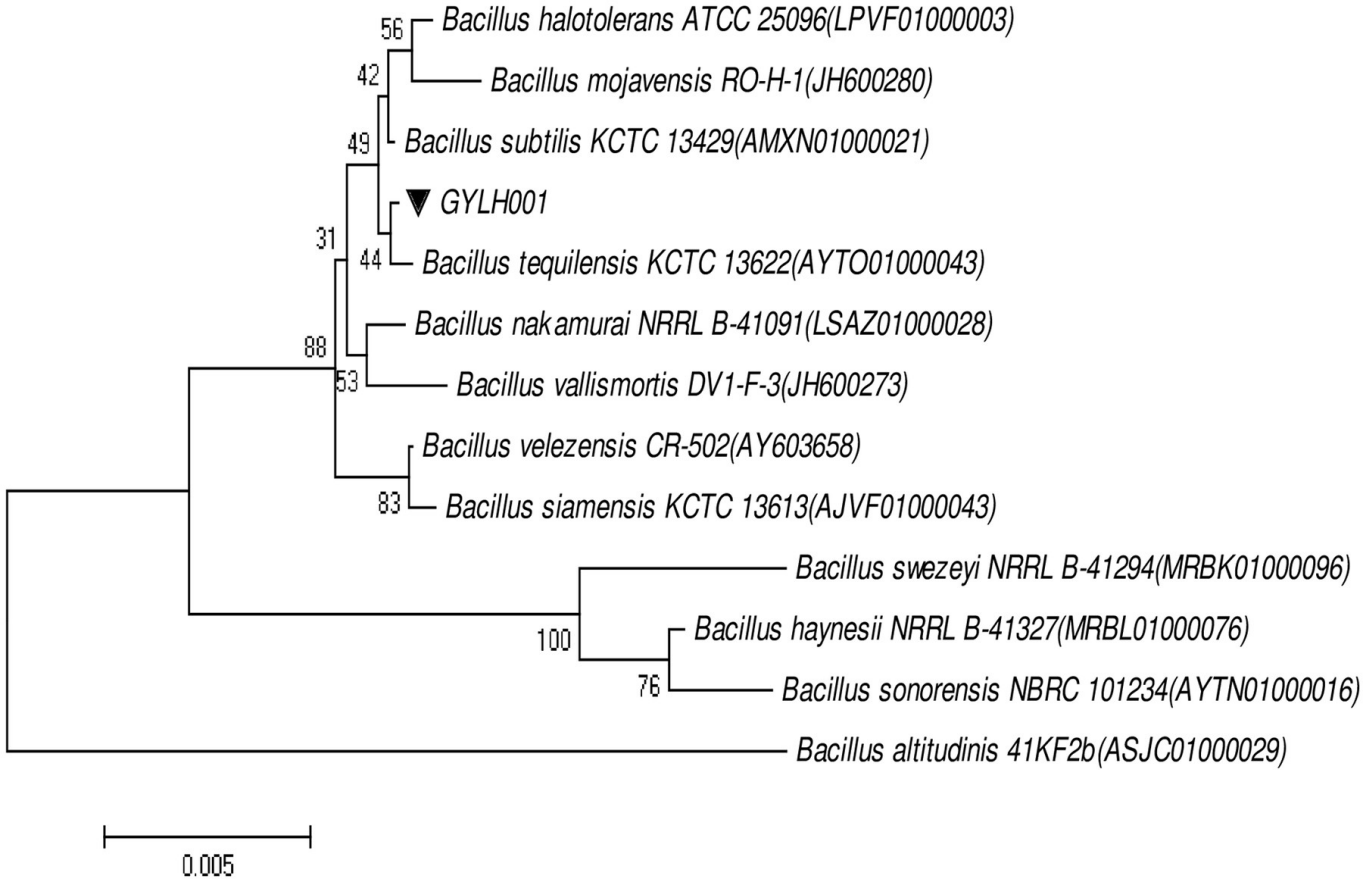
Neighbor-joining phylogenetic tree of GYLH001 based on 16S rRNA sequences analysis.

**Fig 4:**
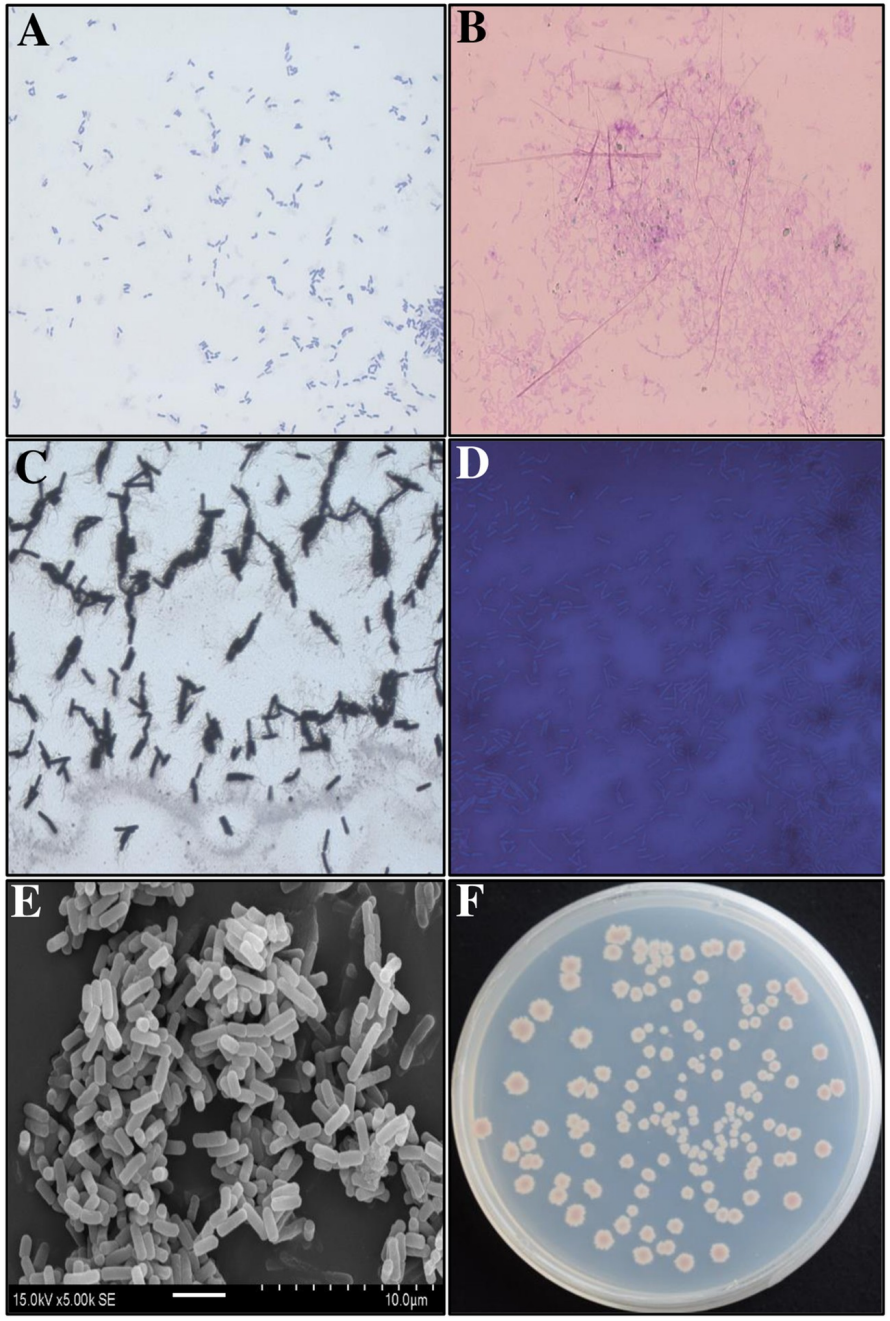
The morphological characteristics of GYLH001. (A) Gram staining. (B) Spore staining observation. (C) Flagella stained observation. (D) Capsule staining observation. (E) Scanning electron microscope observation. (F) Colony morphology; A, B, C, D are viewed under a 1000-fold magnification microscope. Bar = 1cm.

16S rRNA sequence analysis indicated that the strain GYLH001 displayed approximately 99.93% similarity to *B*.*tequilensis* KCTC 13622. A phylogenetic tree displaying the relationship between the strain GYLH001 and other strains was shown in Fig 3. Biochemical and physiological properties of the strain GYLH001 were summarized in Table 1 and the morphological characteristics of GYLH001 were in Fig 4. Gram staining and spore staining were positive, indicating that GYLH001 was gram-positive and contained spores. The capsule staining was negative, which manifested that GYLH001 had no capsule. Scanning electron microscopy (SEM) observations after growth on PDA for 5 days showed GYLH001 was columnar. Combined with the morphological, physiological and biochemical characteristics, GYLH001 was designated as *B*. *tequilensis*. In addition, GYLH001 can grow at a temperature greater than 50°C and grow in 8% NaCl.

**Table 1:** Comparison of physiological and biochemical results of strains GYLH001 and the standard strain of *B*. *tequilensis*.

Items	GYLH001	*B*. *tequilensis* 10b^T^
Shape	Rod	Rod
Anaerobic growth	+	+
Motility	+	+
Oxidase activity	-	+
Decomposition of Tryptophan	+	+
Decomposition of Starch	+	+
Utilization of citrate	+	+
Nitrate reduction to nitrite	-	-
Indole test	-	+
L-Arabinose	+	+
Xylose	+	+
Sorbose	+	+
Methyl red (MR) test	+	ND
Voges-proskauer (VP) tests	+	ND
Growth in 7% NaCl	+	ND
Growth in 8% NaCl	+	ND
Growth in 9% NaCl	-	ND
Growth at 50 °C	+	+

Note: ND, Not determined; +, positive utilization; -, utilization negative. +, good growth;—no growth.

### Effect of pH and temperature

**Fig 5:**
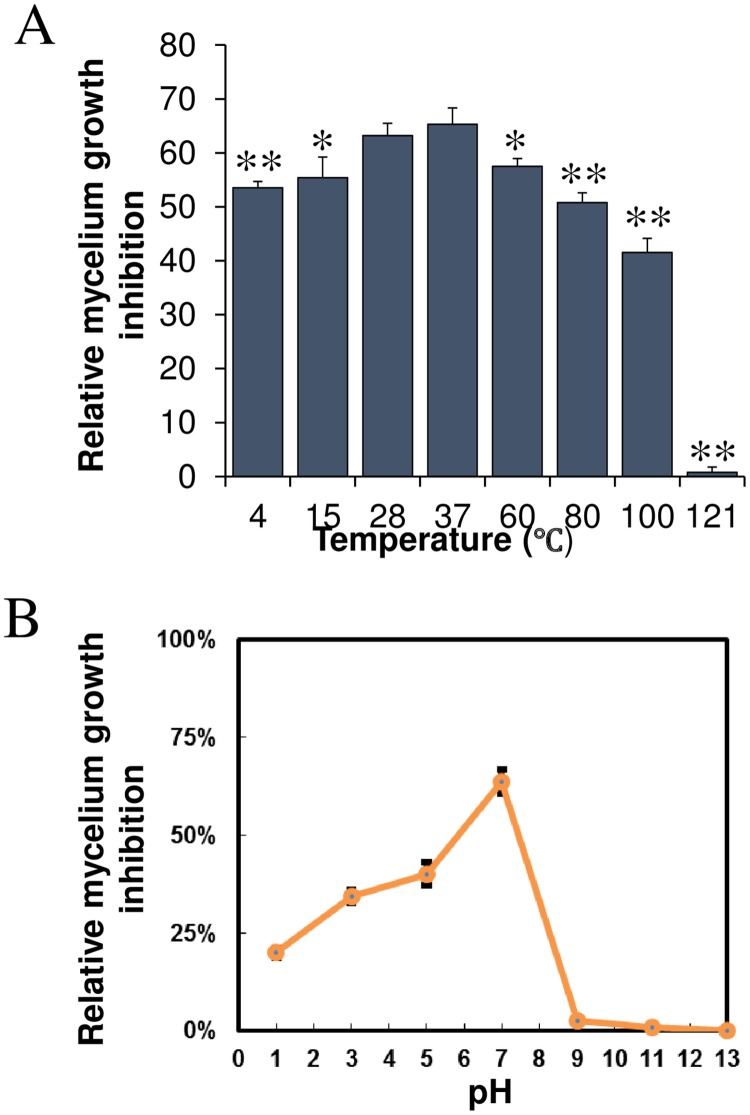
Effects of pH and temperature on the antifungal activity of the sterilized culture filtrate of GYLH001.

The results shown in Fig 5A demonstrated that under the condition of strong acid, neutral and weak alkalinity, sterilized culture filtrate of GYLH001 against *M*. *oryzae* Guy11 was significantly strong. But, with the increase of alkalinity, the inhibitory activity decreased obviously. When PH was 7, the inhibitory activity was strongest. Moreover, the filtrate was shown to be quite thermally stable. When the temperature of the filtrate in the 4°C to 80°C, the inhibition rate can reach to 60%. After 60°C, with the increase of temperature, the inhibition rate decreased obviously. (Fig 5B).

### Growth curve of GYLH001 and its antifungal active

**Fig 6:**
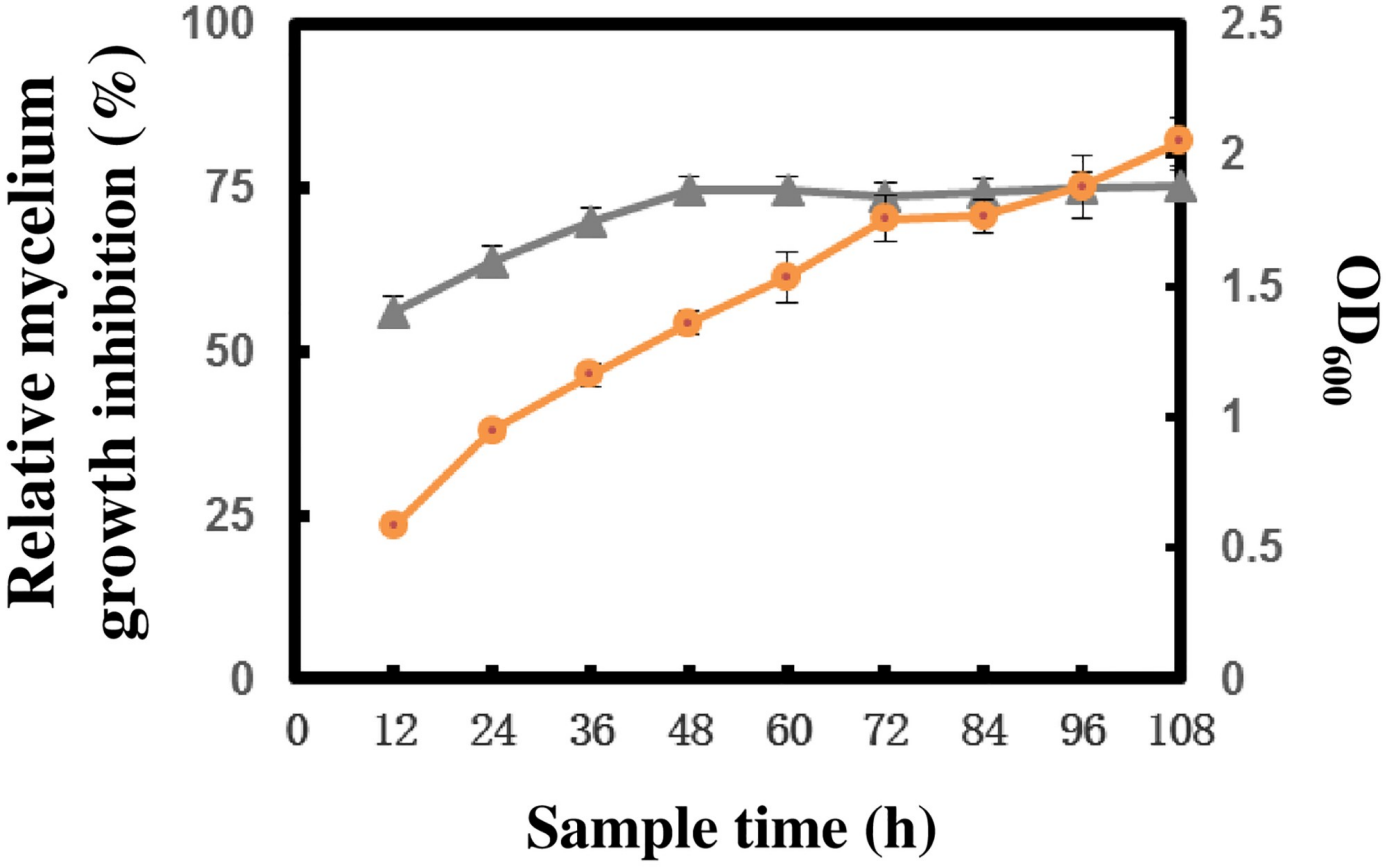
Correlation between cell growth and antifungal activity. ▲ Indicates that antifungal activities of the sterilized culture filtrate of GYLH001 against *M*.*oryzae*. ● means of the growth curve measured by OD_600_.

As shown in Fig 6, the concentration of GYLH001 was positively correlated with the cultivation time. With the increase of cultivation time, the concentration of GYLH001 increased, and the growth trend showed the S curve. At 12 h, GYLH001 entered the logarithmic growth period that GYLH001 reproduced fastest. Moreover, the antifungal activity of the filtrate which sampled at different time intervals was significantly correlated with the growth of strain over the 4-day growth period. The strongest antifungal activity against *M*. *oryzae* was obtained at 48 h after incubation.

### Enzymes and secondary metabolites production by GYLH001

**Fig 7:**
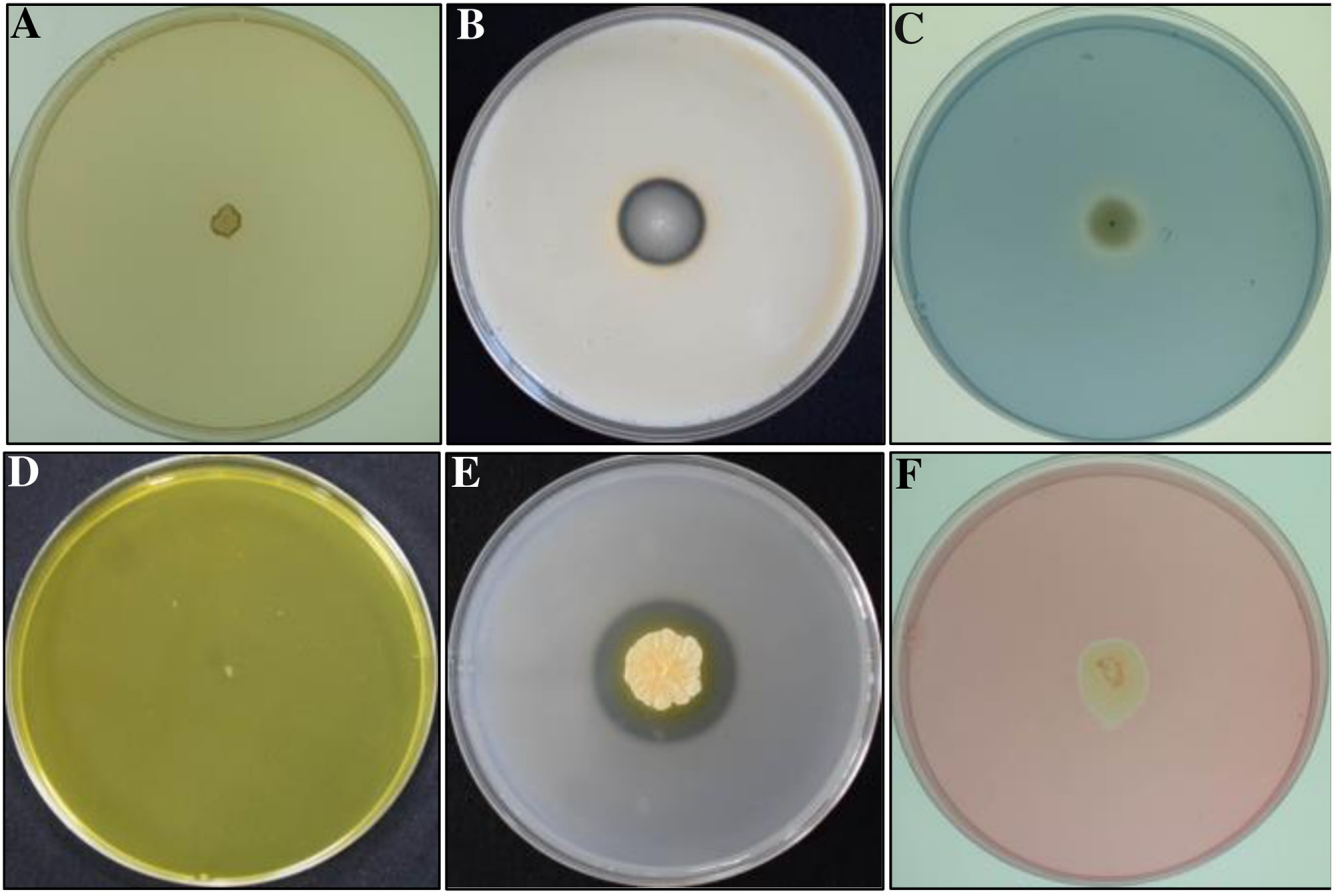
Production of enzymes and secondary metabolites by GYLH001. (A) HCN test. (B) Protease test. (C) Peroxidase test. (D) Chitinase test. (E) Gelatinase assay. (F) Cellulase assay.

For many biological control agents, the active secondary metabolites and fungal cell wall degrading enzymes were a prominent character. So, we found the some enzymes produced by GYLH001 (Table 2). GYLH001 could grow well on protease medium and cellulose medium with a clear zone surrounding the colonies, which indicated that GYLH001 could secrete protease and cellulose. Seen from the gelatin medium, GYLH001 could secret gelatinase with a clear zone surrounding the colony. Although chitinase was an important cell wall degrading enzymes and HCN also could suppress disease, we were unable to found their production by GYLH001 (Fig 7).

**Table 2:** Production secreted by GYLH001.

Production	GYLH001
Chitinase	-
Cellulase	+
Protease	+
Gelatinase	+
ACC deaminase	+
Peroxidase	+
IAA	+
HCN	-

Some secondary metabolites could improve host plant resistance against pathogens by promoting plant growth. GYLH001 could produce plant hormone IAA and ACC deaminase, which could promote plant growth and reduce the harmful effects.

### Evaluation of control efficacy

**Fig 8:**
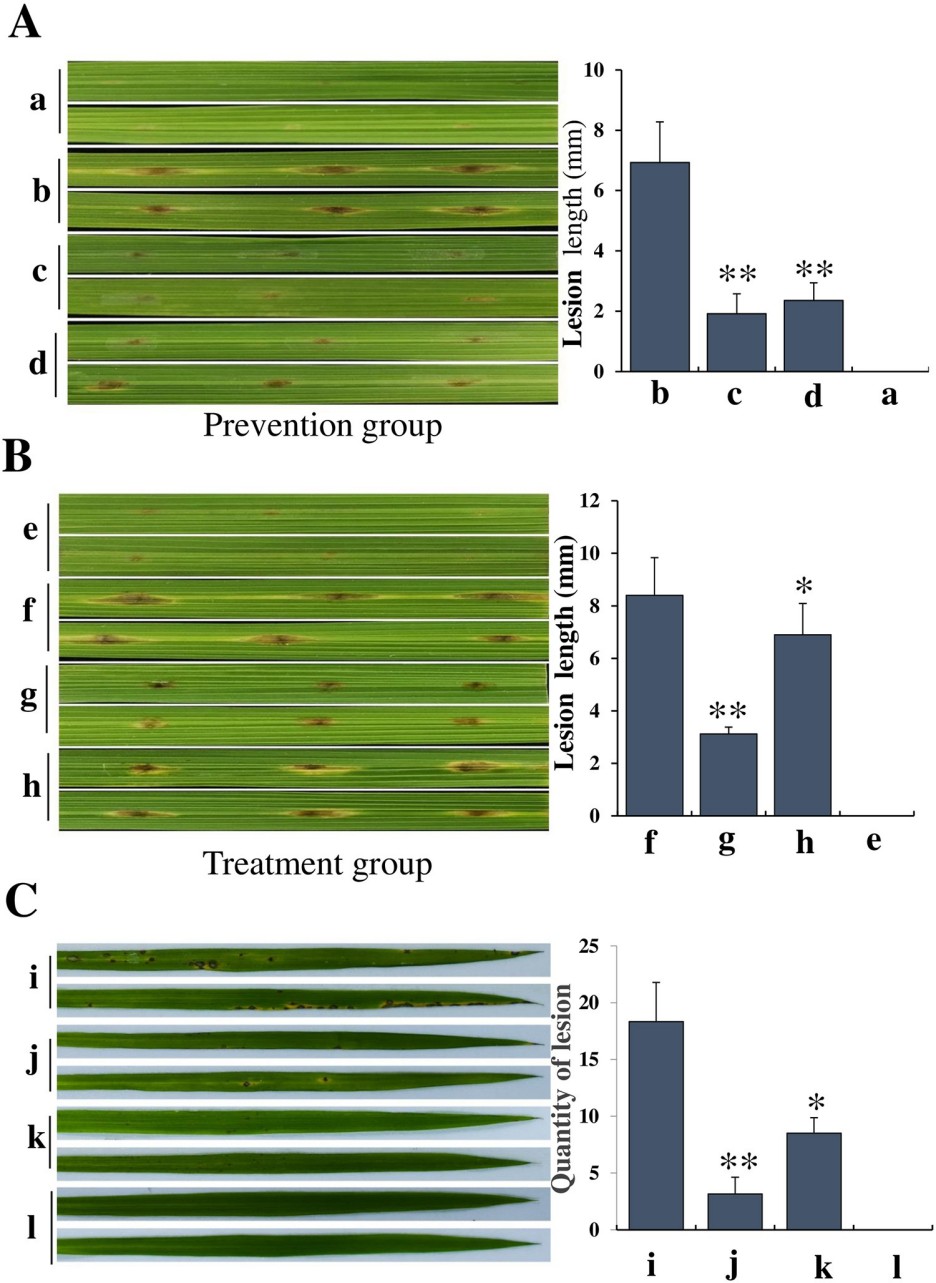
Biocontrol efficiency of GYLH001 against *M*. *oryzae*. (a) Without any treatment. (b) Sterile water was applied to slightly punctured sites of leaves firstly and then spore suspension of *M*. *oryzae* was inoculated. (c) The sterilized culture filtrate of GYLH001 was applied to slightly punctured sites of leaves firstly and then spore suspension of *M*. *oryzae* was inoculated. (d) Bacterial suspension of GYLH001 was applied to slightlypunctured sites of leaves firstly and then spore suspension of *M*. *oryzae* was inoculated. (e) Without any treatment. (f) Spore suspension of *M*. *oryzae* was inoculated to slightly punctured sites of leaves firstly and then sterile water was applied to the same site. (g) Spore suspension of *M*. *oryzae* was inoculated to slightly punctured sites of leaves firstly and then the sterilized culture filtrate of GYLH001 was applied to the same site. (h) Spore suspension of *M*. *oryzae* was inoculated to slightly punctured sites of leaves firstly and then the bacterial suspension of GYLH001 was applied to the same site. (i) Spraying spore suspension of *M*. *oryzae* after spraying the sterilized water. (j) Spraying spore suspension of *M*. *oryzae* after spraying the sterilized culture filtrate of GYLH001. (k) Spraying spore suspension of *M*. *oryzae* after spraying 0.75g / L tricyclazole. (l) Without any treatment; *Difference is significant at the 0.05level, while **difference is significant at the 0.01 level.

Disease control efficacy of GYLH001 against rice blast was shown in Fig 8. In the Fig 8A, compared with control group, lesion diameter of leaves of prevention group was obviously smaller than control group. Similarly, in the treatment group, so it is. However, the results indicated that GYLH001 displayed significant preventive effect, which was higher than curative effect. It exhibited weak curative activity against the rice blast. In the Fig 8B, compared with i group, quantity of lesion of j group and k group was significantly reduced. This phenomenon indicated that the sterilized culture filtrate of GYLH001 had the control effect of rice blast the same as tricyclazole.

### Isolation of the active substances

Through elution of column chromatography, the mixture was divided into 11 gradients. The fraction 5 which was eluted with 100% methanol displayed strong activity and was chosen for further purification.

**Fig 9:**
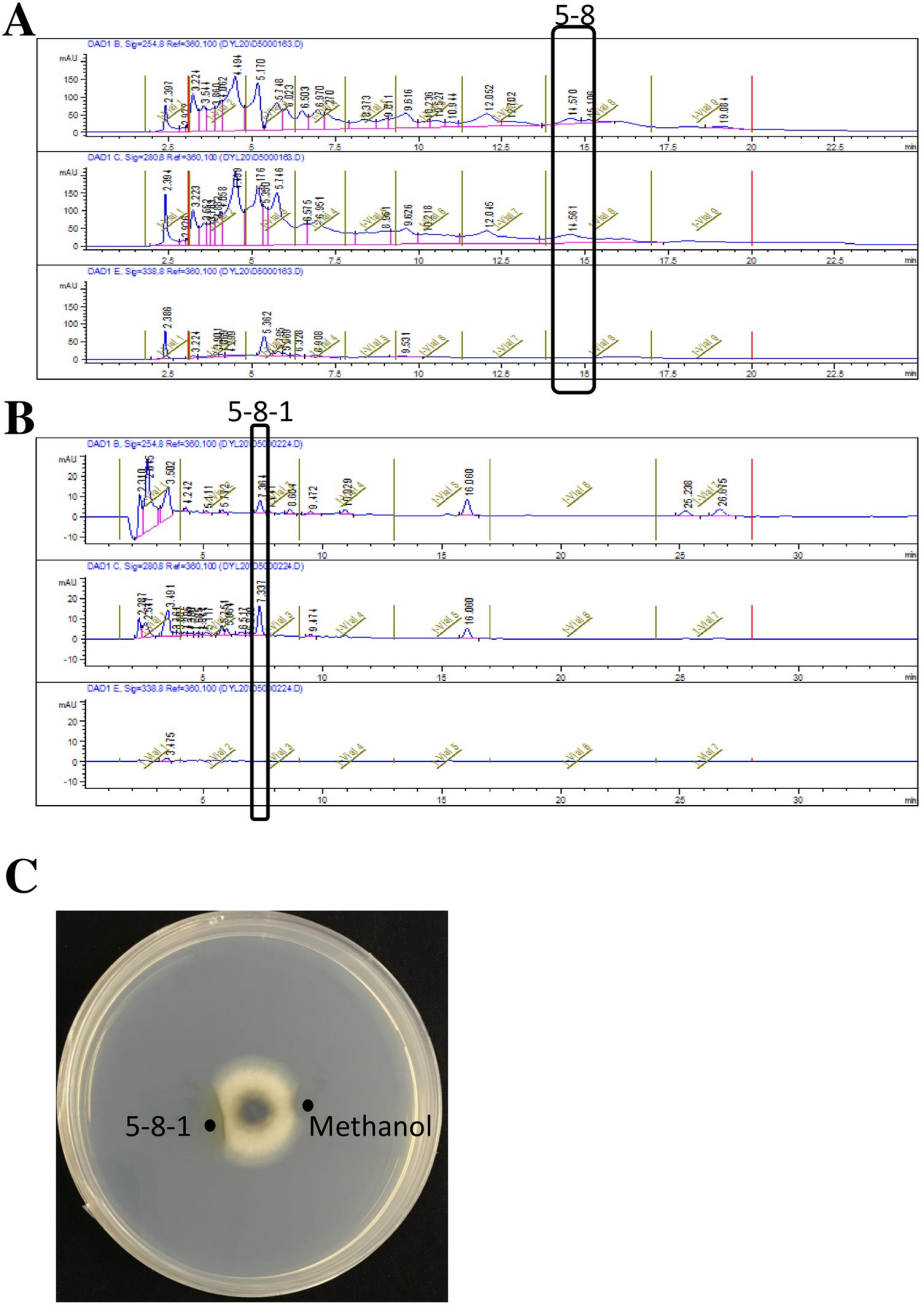
Purification of antifungal substances by HPLC. (A) Chromatogram of ODS-3. (B) Chromatogram of Waters C18. (C) Activity detection of fraction 5-8-1.

As shown in the Fig 9A, the fraction 5 was divided into nine fractions according to the peak time through the preliminary separation by HPLC (Inersil ODS-3, 0.8 mL/min, MeOH-H_2_O, 67:33). The retention time is 14–15.5 min for fraction 5–8 which displayed strong activity. In the Fig 9B, the fraction 5–8 was further separated (Waters C18, 0.8 mL/min, H_2_O-Acetonitrile, 57:43). When the retention time is 7–7.8 min, the fraction 5-8-1 displayed strong activity (Fig 9C).

**Fig 10:**
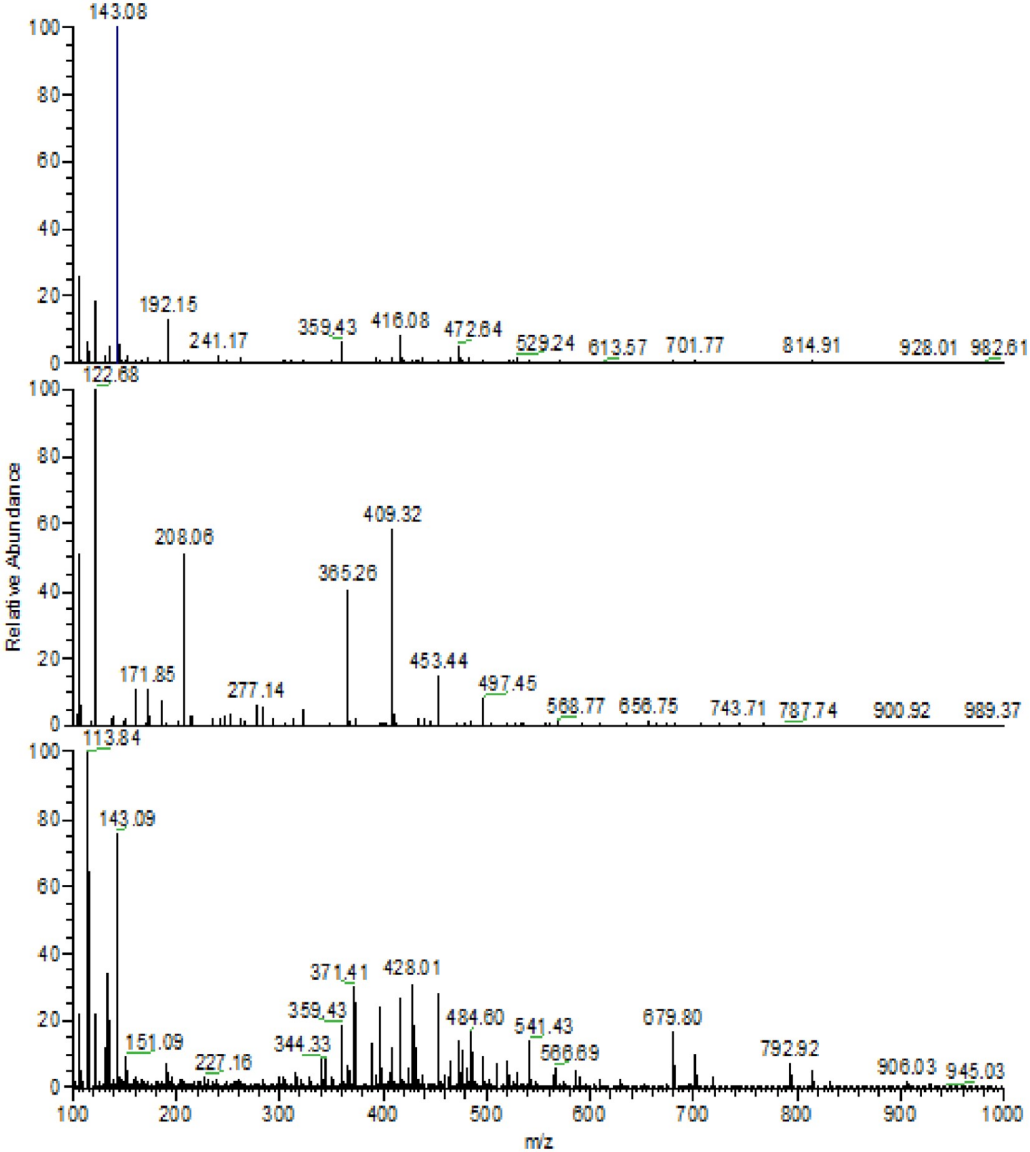
Mass spectrum of active substance 5-8-1.

Detection by mass spectrometry, the molecular weight of the substance is 364.26 (Fig 10).

## Discussion

In the nature, there are a lot of antagonism among different species. For a long time, people have been committed to taking full advantage of antagonism to apply to many fields such as agricultural defense and medical domain. Plant fungal diseases are difficult to control, which makes it possible to lead to huge economic losses. Thus, strategies to ensure that plants avoid to fungal infection should be developed, especially by the development of biological control methods [34].

A number of plants from the apiacease, such as *Angelica dahurica*, show cytotoxicity, antitumor and are against pathogenic microorganism. In our current study, several endophytic strains have been isolated from the *Angelica dahurica*. GYLH001 was one of the most active endophytic strains which can inhibit the growth of plant pathogens *M*. *oryzae*. The sterilized culture filtrate of GYLH001 significantly inhibited growth of *M*. *oryzae*. The strong antifungal activity and lack of toxicity towards rice showed the potential of the strain for the application in rice blast protection. In the present study, based on the physiological, biochemical characteristics, and 16S rRNA sequence analysis, GYLH001 was identified as *B*. *tequilensis*. To the best of our knowledge, this is the first isolation of endophytic *B*. *tequilensis* from *Angelica dahurica*.

Due to the production of cell wall degrading enzymes and other antifungal natural products, Many species of strains, especially those belonging to the *Bacillus*, can inhibit the growth of phytopathogenic fungi [35]. We find that GYLH001 can produce extracellular enzymes, which makes it possible to have the capacity to demonstrate multiple mechanisms against *M*. *oryzae*. First, chitinase, cellulase and protease might cause an abnormal hyphal morphology of pathogens [36]. Therefore, cellulase and protease secreted by GYLH001 can cause the hyphal deformation and growth suppression of *M*. *oryzae*. Second, IAA and ACC deaminase secreted by GYLH001 can promote plant growth by reducing the adverse effects of ethylene. As we know, plant growth promoters might improve plant disease resistance indirectly. Maybe, it could be a result of the antagonism of *Bacillus* in the protection of host rice against pathogens by promoting the growth of rice. In addition, further studies aiming to identify the compounds responsible for the antifungal activity of GYLH001 are in progress in our laboratory.

Through the stability assessment experiment, the results show that the active metabolites have good stability for the strong acid and high temperature. It has laid a good foundation for the future of biological control against plant disease. In particular, the stability of high temperature of active metabolites is an advantage of biological pesticide research and development, which overcomes the disadvantages of saving of traditional biological pesticide.

At the given fermentation condition, *B*. *tequilensis* GYLH001 enter the logarithmic growth period after 12 h of cultivation. The strongest of antifungal activity was obtained during the stationary phase 48 h after inoculation. With the increase of inoculation time, antifungal activity is almost constant. In the previous research, at the end of the exponential growth phase, the antibiotics usually starts to be produced. It reaches maximum concentration after cell growth having ceased and this is in agreement with the production kinetics of the strain GYLH001.

By evaluation of control efficacy, we can find that GYLH001 significantly inhibits the growth of rice blast. But, we find that the preventive effect is better than the therapeutic effect. To our knowledge, the present chemical fungicides are only effective for preventing and not effective for curing rice blast [22]. The result obtained with GYLH001 shows a similar pattern. So we deduce that GYLH001 might be effectively conducive to the plant resistance of disease by secreting antifungal metabolites and growth promoters.

Although we obtained a pure substance with strong bacteriostatic effect by continuous separation and purification, its molecular weight was 364.26. However, due to the extremely unstable metabolic process, it is impossible to collect sufficient quantities for nuclear magnetic resonance examination and infer its structure. In future studies, we will optimize the separation and purification of each fraction and obtain the structure of the substance as soon as possible for subsequent studies.

In a word, *B*. *tequilensis* GYLH001 appears to be a promising candidate as a biological control agent for rice blast in agricultural applications.

## Supporting information

S1 Table 16S rRNA base sequence of GYLH001 (1542bp). (DOCX)
